# Sonochemical Effects on the Preparation, Structure and Function of Gliadin-(−)-Epigallo-Catechin 3-Gallate Conjugates

**DOI:** 10.3390/foods12071376

**Published:** 2023-03-24

**Authors:** Jiaxing Cao, Ning Xu, Jianhao Zhang, Guozhi Zhang, Yu Zhang

**Affiliations:** 1 College of Food Science and Engineering, Henan University of Technology, Zhengzhou 450001, China; 2 College of Food Science and Nutritional Engineering, China Agricultural University, Beijing 100083, China; 3 College of Food Science and Technology, Shanghai Jiao Tong University, Shanghai 201100, China

**Keywords:** gliadin, ultrasound, ultrasound-assisted free radical reactions, protein structures, functional properties

## Abstract

It is essential to understand the mechanism of action of ultrasound synergistic free radical oxidation to promote covalent reactions between proteins and polyphenols. (−)-epigallo-catechin 3-gallate (EGCG) with rich bioactivity could be used to increase the functional properties of cereal protein—gliadin (GL). This study systematically explored the role of ultrasound treatment (US) on the binding mechanisms of GL and EGCG. Electrophoresis and high-performance liquid chromatography (HPLC) confirmed the greater molecular mass of the covalent complexes in the ultrasound environment. Quantitative analysis by the phenol content revealed that the ultrasound environment increased the EGCG content in the covalent complex by 15.08 mg/g of protein. The changes in the spatial structure of the proteins were indicated by Fourier infrared and ultraviolet spectroscopy. Additionally, scanning electron microscopy (SEM) and atomic force microscopy (AFM) found that US disrupted the aggregation of GL and the clustered structure of the covalent complexes. The results demonstrated that the water solubility of ultrasonic conjugates was significantly increased by 8.8–64.19%, the digestion rate was more efficient, and the radical scavenging capacity was twice that of GL. This research contributes to the theoretical basis for broadening the application of polyphenols in modifying protein.

## 1. Introduction

Wheat is used as a major food crop worldwide, accounting for approximately 30% of the total global production of cereal commodities. Although dietary habits vary slightly around the world, wheat-based foods are still used as one of the staple foods for most people. Gluten protein is the major storage protein in wheat, accounting for about 80–85% of wheat protein [1]. Gliadin (GL) is the main component of gluten protein, which can be divided into four main groups based on gel mobility: α-, β-, γ- and ω-. GL is often used as a carrier particle for active substances as well as for stabilizing emulsions due to its amphiphilic nature and surface activity [2]. Additionally, GL can be used as a plasticizer to weaken the interaction between gluten chains and thus increase the dough’s viscosity during the production of wheat products [3,4]. As the main hydrophobic storage protein among wheat proteins, it is difficult for GL to meet the demand in practical production because of its poor solubility. Therefore, high temperature and pressure, ultrasound, and plasma are often used to improve its functional characteristics [5,6]. Alternatively, a substance (polyphenols, polysaccharides, etc.) could be added to modify the protein to improve the physicochemical properties of the protein using interactions.

Owing to their excellent pharmacological effects and antioxidant properties, catechins (CA) are often used in the modification of food biomolecules. In this regard, (−)-epigallo-catechin 3-gallate (EGCG), as one of the catechins with high bioactivity, is often used in food, drug and cosmetic applications. As reported, EGCG exhibited good anti-allergic, antioxidant and anti-inflammatory properties [7]. EGCG can covalently react with active sites in GL such as amino (-NH_2_) or sulfhydryl (-SH) groups in alkaline, free radical, enzymatic and other specific environments [8].

However, free radical and alkaline grafting, used commonly to construct covalent reactions, are currently inefficient, which limits the application of covalent technology in the food industry. Research had found that US promoted the dissociation of water molecules to produce OH^-^ [9], which is more conducive to the formation of protein radicals. Furthermore, the structural transformation of proteins in the ultrasound environment might expose more sites of reaction (tyrosine, proline, etc.), thus being more favorable for the formation of covalent bond. Research on physical techniques to enhance polyphenol modifications has emerged; however, it mainly focuses on animal proteins, with less research relating to plant proteins [10,11]. Whether or not the covalent binding of plant proteins to dietary polyphenols could be promoted by ultrasound-assisted means deserves our further study.

In order to improve the sustainability of food production, and to develop phytoproteins with excellent processing properties, this research, on the covalent reaction mechanism of GL/EGCG based on an ultrasound environment, was explored. Firstly, the conjugation efficiency of polyphenols was compared between conventional free radical and sonicated radical environments. Then, the structures of the ultrasound, covalent and ultrasound-covalent samples were analyzed for potential mechanisms of US-assisted covalent reactions. Lastly, the processing properties, antioxidant properties and digestibility of the samples were evaluated. These results provide new a theoretical basis and technical guidance for the study of cereal protein–polyphenol conjugates.

## 2. Materials and Methods

### 2.1. Materials

Wheat flour was provided by Henan Academy of Sciences (Zhengzhou, China). EGCG (purity ≥ 98%) was purchased from Solarbio Co. Diammonium 2,2′-azino-bis (3-ethylbenzothiazoline-6-sulfonate) (ABTS), 1,10-diphenyl-2-picrylhydrazyl (DPPH), bovine serum albumin (BSA), and Folin–Ciocalteu phenol reagent were purchased from Sigma Chemical Co. (St. Louis, MO, USA). Sodium dodecyl sulfate-polyacrylamide gel electrophoresis kit was purchased from epizyme biomedical technology Co. (Shanghai, China). All other chemicals were of analytical grade.

### 2.2. Preparation of Gliadin

The wheat flour was mixed with ether (*w*:*v* = 1:3) and stirred at room temperature for 4 h. Then, the precipitate was collected by centrifugation at 4500 r/min for 15 min. The defatted wheat flour was dispersed into 65% ethanol in the ratio of 1:10 (*w*/*v*), and stirred at room temperature for 12 h. The supernatant was collected by centrifugation at 5000 r/min for 20 min. Finally, the collected supernatant was distilled at 40 °C under reduced pressure to remove the ethanol. The concentrate was freeze-dried to obtain GL powder, and it was stored at −40 °C.

### 2.3. Preparation of Conjugates

The combined treatment of GL was performed to investigate the reinforcing effect of US on the covalent reaction. GL was treated in 4 different treatments—GL: without EGCG and US; US-GL: with US but without EGCG; GLE: GL with EGCG but without US; US-GLE: GL with EGCG and US.

Briefly, ascorbic acid/H_2_O_2_ was added to a GL solution and EGCG was added for covalent reaction after stirring at room temperature for 30 min. Ultrasonic treatment was performed at the same moment, setting the power to 480 w and treating at room temperature for 2 h. Dialysis was performed for 72 h after the reaction, and the dialysis solution was changed every 6 h. US-GL was performed according to the above ultrasonic conditions but without the addition of ascorbic acid/H_2_O_2_ and EGCG to the solution.

### 2.4. Electrophoresis of Proteins

SDS-PAGE was performed as described in the kit (Epizyme biomedical technology Co., Ltd., Shanghai, China). 12.5% polyacrylamide gels were prepared. Briefly, the lower gel solution was mixed with the lower gel buffer (*v*:*v* = 1:1), and added to the gel-making tank. The preparation of the upper layer gel was similar to the preparation of the lower layer gel. Electricity electrophoresis experiments were started after the gel solidified. The sample buffer was mixed with the sample and boiled, and then the supernatant was aspirated by centrifugation. Thereafter, 15 μL of supernatant was added to the sample tank of the upper gel layer. The voltage was adjusted to 60 V so that the bromophenol blue band migrated to the separation gel. Finally, the voltage was adjusted to 120 V again until the end of electrophoresis.

### 2.5. High Performance Liquid Chromatography (HPLC)

HPLC was performed according to the method of Men et al. [12]. The method of HPLC 1260 (Agilent Co. Ltd., Santa Clara, CA, USA) and Protein-Pak 60 (7.8 mm × 300 mm, Waters) were used with modifications to determine the molecular weight distribution. Specifically, the sample solution (5 mg/mL) was filtered through a 0.45 μm filter membrane, and then transferred to a 2 mL injection vial. The column was eluted with acetonitrile solution containing 0.1% TFA (acetonitrile concentration of 30%) at a flow rate of 0.5 mL/min. The wavelength of the detection sample was 280 nm.

### 2.6. Conjugates Polyphenol Equivalent Detection

The phenol content of the samples was determined using the Folin–Ciocalteu method according to Wu et al. [13]. A mixture (6 mL) containing 1 mL of different protein solutions after various modifications and 5 mL of purchased Folin–Ciocalteu reagent (0.25 mol/L) was placed at room temperature for dark reaction (5 min, 25 °C). Then, 0.8 mL of the configured Na_2_CO_3_ solution (7.5%, *w*/*v*) was added to the original solution for the reaction over 60 min. After centrifugation at 4500 r/min for 20 min, 200 μL of supernatant was taken to the enzyme plate for the measurement of the absorbance (760 nm). The EGCG content of the binding of different GLs was calculated using the standard curves of EGCG. The grafting efficiency (%) was determined as polyphenol content in the reactants after dialysis/polyphenol content in the reactants before dialysis × 100%.

### 2.7. Determination of Free Sulfhydryl Groups (-SH)

The determination of -SH was performed as described by Xu et al. [14]. Briefly, 30 μL of DTNB (4 mg DTNB was dissolved in 1 mL of 50 mM Tris-HCl buffer containing 1 mM EDTA) was added to 3 mL of GL solution and mixed rapidly. Then, it was protected from light at room temperature for 15 min. The absorbance was measured at 412 nm. The sulfhydryl thiol content (nmol/mg) was calculated by Equation (1)

(1) $$SH=73.53\times A_{\left(412\right)}\times \frac{D}{C}$$

where *A*_(412)_, *D*, and *C* represent the absorbance at 412 nm, the dilution factor, and concentration, respectively.

### 2.8. Determination of Free Amino Acid Groups (NH_2_)

The determination of free amino groups was carried out as previously reported [15]. Trinitro-benzene-sulfonic acid (TNBS) was used for this determination. To be specific, 400 μL of TNBS solution (0.1%, *w*/*v*) was added to 500 μL of 0.1 mg/mL sample solution (samples were dissolved with PBS at pH 8.2). Subsequently, the mixture was reacted in a water bath protected from light (50 °C, 1 h). 800 μL HCL (100 mM) was added to terminate the reaction, and then the absorbance at 340 nm was measured. The lysine standard curve was determined and established using the same method. Then, the free amino acid content was calculated from the lysine standard curve.

### 2.9. Fourier Transform Infrared (FT-IR) Spectroscopy

The lyophilized samples were mixed with KBr at a ratio of 1:100 (*w*/*w*), ground and pressed. FTIR spectroscopy (Bruker, Billerica, MA, USA) of the samples was conducted in accordance with Zhang et al. [16]. The FT-IR scan range was 400–4000 cm^−1^ with a resolution of 4 cm^−1^. The scan was performed 32 times with KBr as the background.

### 2.10. Circular Dichroism Spectrum (CD)

The samples were assayed as previously reported [17]. The CD spectrum was collected in the range 190–260 nm. The sample was dissolved in PBS (pH: 7.0) at a concentration of 0.2 mg/mL.

### 2.11. UV-Vis Spectrum (UV)

Protein samples were dissolved in 65% ethanol (0.25 mg/mL). UV-2600 (SHIMADZU Inc., Kyoto, Japan) was adjusted to a slit width of 2 nm with a wavelength interval of 1 nm. The UV absorption spectra of samples in the range of 190–450 nm were collected with 65% ethanol as a blank.

### 2.12. Scanning Electron Microscope (SEM)

A suitable number of samples was sprayed with gold for microstructure acquisition by SEM (S-3400; Hitachi Inc., Kyoto, Japan). SEM acceleration voltage was set to 20 kV, and to magnifications of 5000 times.

### 2.13. Atomic Force Microscope (AFM)

The microstructure of samples was observed using AFM according to the method described by Li et al. [18]. The sample solution (0.05 mg/mL) was dropped onto a mica sheet and dried at room temperature for 2 h. Images were collected using a nan-probe cantilever tip in percussion mode (frequency: 50–100 Khz). Nanoscope Analysis version 1.5 was used for image analysis.

### 2.14. Surface Hydrophobicity (SHY)

The SHY of samples was expressed in terms of the amount of bromophenol blue (BPB) bound [19]. Briefly, 1 mL of sample (2.5 mg/mL) was mixed thoroughly with 1 mL of PBS (PH = 7) and 200 μL of BPB. The absorbance of the supernatant obtained by centrifugation at 595 nm was measured using UV-2600. BPB binding was calculated by Equation (2)

(2) $$BPB\;binding\;\left(\mu g\right)=200\;\mu g\times \frac{\left(A_{0}-A_{N}\right)}{A_{0}}$$

where *A*_0_ and *A_N_* represent the PBS blank and sample absorbance, respectively.

### 2.15. Water Solubility

The solubility of protein was determined using the approach described by Zhang et al. [20] with some slight modifications. Samples were dispersed by deionized water (20 mg/mL) and processed in a constant temperature water bath shaker for 30 min. The mixture was centrifuged (10,000× *g* for 15 min) to collect the supernatant, and then the absorbance of the supernatant was measured at 595 nm. The protein concentration in the supernatant was calculated from the bovine serum protein standard curve.

### 2.16. Water and Oil Holding Capacity (Whc and Ohc)

The method described by Zhang et al. [21] with modifications was used to determine the water-holding and oil-holding properties of the GL. The sample was mixed with 25 mg of water/oil in a pre-weighed centrifuge tube and left to stand at room temperature for 30 min. After centrifugation at 1500× *g* for 10 min, the supernatant was poured off and the excess water/oil was removed using filter paper and weighed.

### 2.17. Emulsifying and Emulsifying Stability

Emulsification properties were determined as previously reported [22]. The sample solution (2 mg/mL) was mixed with soybean oil at 3:1 (*v*/*v*) and then blended for 2 min using a high-speed homogenizer (10,000× *g*). An amount of 50 μL of the emulsion was mixed with 5 mL of SDS (0.1% (*w*/*v*)), and the absorbance was measured at 0 and 10 min at 500 nm. The emulsifying activity index (EAI) and emulsion stability index (ESI) were calculated by Equations (3) and (4):

(3) $$EAI=\left(m^{2}/g\right)=\frac{\left(2\times 2.303\times A_{0}\times D\right)}{\left(N\times C\times 10000\right)}$$

(4) $$ESI\;\left(\min\right)=A_{0}\times \frac{T}{\left(A_{0}-A_{10}\right)}\times 10$$

where *A*_0_ and *A*_10_ indicate absorbance at 0 and 10 min, respectively; *D* is the dilution factor; *N* is the volume fraction; *C* is the protein concentration; *T* is the stabilization time.

### 2.18. Antioxidant Performance Analysis

The determination of DPPH and ABTS^+^ scavenging capacity was performed as described by He et al. [23] with modifications. The free radical scavenging activity (Trolox/mg) of the samples was calculated using the Trolox standard curve. Briefly, the sample solution was mixed with DPPH at 1:1 (*v*/*v*), and the absorbance value was measured at 517 nm after 1 h of reaction at room temperature with protection from light. The sample solution was mixed with ABTS+ at 1:2 (*v*/*v*), and the absorbance value was measured at 734 nm after 1 h of reaction at room temperature with protection from light.

Fe^3+^ reduction antioxidant power (FRAP) was carried out according to the following method. An amount of 10 μL of sample solution (0.5, 1.0, 1.5, 2.0, 2.5 mg/mL) was mixed with 300 μL of FRAP working solution (0.3 M sodium acetate (pH = 3.6), 10 mM 2,4,6-Tripyri-dyltriazine and 20 mM FeCl_3_ mixed at a ratio of 10:1:1 (*v*/*v*/*v*) followed by dark reaction in a water bath at 37 °C for 2 h). The absorbance value was measured at 593 nm after 30 min of reaction at 37 °C with protection from light.

### 2.19. Simulation of Gastrointestinal Fluid Digestion

Simulated digestion tests were performed as described by Li et al. [24] with some adjustments. The sample solution (2 mg/mL) was prepared with 45 mM of NaCl solution, stabilized at 37 °C for 15 min before adding pepsin (pepsin: substrate = 0.03 (*w*/*w*)). The samples were collected at 60 min and 120 min of pepsin digestion (SGF-60 min and SGF-120 min) while adding NaHCO_3_ (2 M, 50 μL) to terminate the reaction. Trypsin (2.5 g) and porcine bile extract (10 mg) were dissolved in 25 mL NaHCO_3_ to adjust the pH to 7 to simulate intestinal fluid digestion. Samples from the terminated stage of gastric digestion were continued with intestinal fluid digestion (trypsin: substrate = 0.025) at 37 °C in a constant temperature shaker. The samples were collected for 30 min and 60 min of trypsin digestion (SIF-30 min and SIF-60 min) and immediately placed in a 100 °C water bath for enzyme inactivation. The samples were assayed for protein concentration using the BCA kit with protein digestibility calculated by Equation (5):

(5) $$Digestibility\;\left(\%\right)=\frac{C_{0}-C_{n}}{C_{0}}\times 100$$

where *C*_0_ and *C_n_* represent the concentration of protein without and after the digestion reaction, respectively.

### 2.20. Date Analysis

Data were processed mainly by SPSS 26.0 and origin Pro and expressed as the means ± standard deviations of triplicate determinations. Gel electrophoresis and AFM data were analyzed by Image Lab and NanoScope Analysis 1.9, respectively.

## 3. Result and Discussion

### 3.1. Changes in Protein Group Content

**Figure 1:**
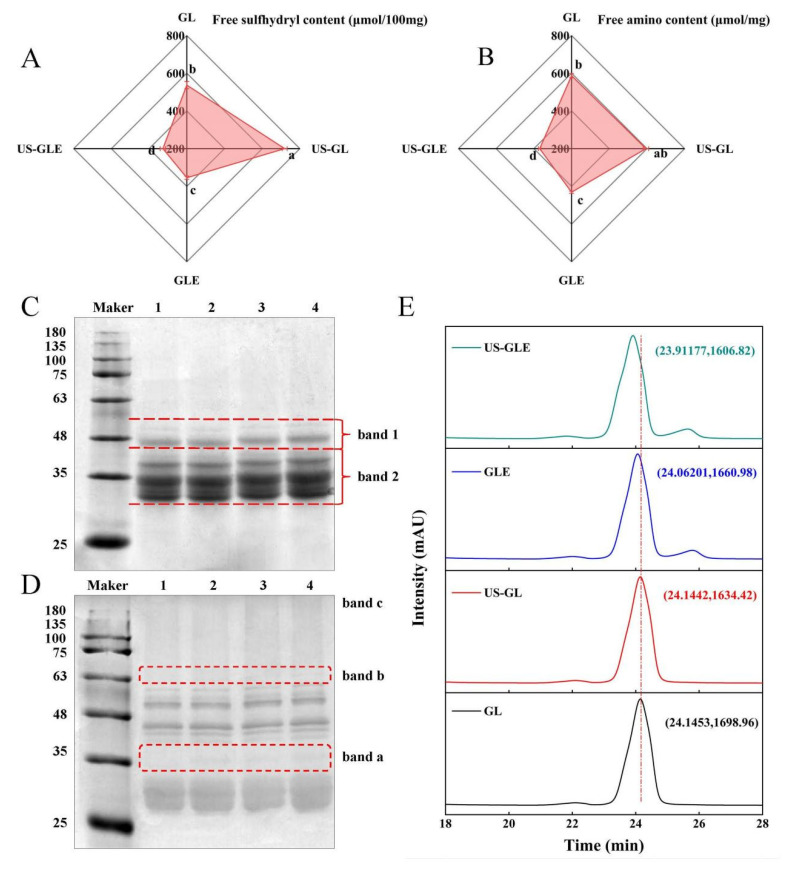
Free -SH (**A**), free -NH_2_ (**B**), SDS-PAGE (**C**), non-reducing SDS-PAGE (**D**) and HP-LC (**E**) analysis of GL, US-GL, GLE, and US-GLE. The electrophoretic bands of GL, US-GL, GLE, and US-GLE are shown in lanes 1–4 in (**C**) and (**D**), respectively. x and y in (**E**) indicate HP-LC peak retention time of the sample and UV absorption peak intensity, respectively. Values with different letters (a–d) in the figure indicate significant differences (*p* < 0.05).

The process of free radical-induced covalent binding was essentially the formation of covalent bonds between the side chain groups of the oxidized proteins with the neighboring and opposite positions of the phenolic hydroxyl groups of the polyphenols [25]. The variation in the content of the side chain groups responded to the degree of covalent binding of polyphenols to proteins. As shown in Figure 1A,B, the -SH and -NH_2_ content in GL was 537.29 nmol/100 mg and 587.25 nmol/mg, respectively. There was a significant increase in -SH and NH_2_ in GL after sonication, in which the -SH increased by 182 nmol/100 mg. As they were subjected to the high sheer force of US, disulfide bonds that maintained the spatial structure of the protein were broken. The -SH and -NH_2_ contents in GLE were reduced to 354.24 nmol/100 mg and 430.94 nmol/mg, respectively, which corresponded to the consumption of the covalent reaction. Meanwhile, the transformation of the protein spatial structure during the covalent reaction may have also masked the -SH and -NH_2_. The noteworthy point was that the -SH and -NH_2_ value of US-GLE was reduced substantially. Additionally, the reducing rate of the -SH and -NH_2_ intensity of the US-GLE group (54.64%, 38.02%) was significantly higher than that of the GLE group (34.03% and 26.62%), implying that the synergistic action of cavitation bubbles and free radicals promoted the consumption of the GL hydrophobic groups. Conformational stretching and partial unfolding of GL were responsible for leading to a sensitive state of protein during US. Free radical oxidation and the repeated action of cavitation bubbles strengthened the denaturation of the GL structure. In this regard, EGCG preferred to bind covalently to GL in a sensitive state.

### 3.2. SDS-PAGE Analysis of Protein

The changes in the subunit migration rate and the degree of aggregation of protein molecules were observed by SDS-PAGE. As shown in Figure 1C, there were no significant changes in the subunits of GL after ultrasound pretreatment, since the energy provided by ultrasound was not sufficient to break the peptide bonds, which was consistent with the findings of Zhang et al. [26]. GL did not produce small molecular subunits and large molecular aggregates after covalent reaction/US. However, the subunits of both GLE and US-GLE showed significant shifts, and the migration rate of protein bands on the gel was slowed down. These changes indicated the increase in the relative molecular mass of GL after covalent binding with EGCG. The slower subunit migration rate of US-GLE compared to that of GLE was probably attributable to the reinforcing effect of US. During free radical oxidation, US promoted the structural transformation of GL via the strong shearing action, contributing to the exposure of reaction sites. Meanwhile, the cavitation effect increased the content of OH^-^ in the reaction environment [27]. These radicals can act on the amino acids in the GL chain to contribute to covalent linkage with EGCG. As shown in Figure 1D, the non-reducing SDS-PAGE analysis revealed that US-GL, GLE and US-GLE were shown at new bands, at “band a” and “band b”, which could have been due to the disulfide bond cross-linking of small molecular subunits. In essence, the -SH of the side chain was oxidized, and then the disulfide bond was formed between GL, this process being mainly driven by OH^-^ from cavitation effects and the H_2_O_2_/hydrogen peroxide redox reaction. Furthermore, GLE and US-GLE were deepened at “band c”, indicating that the covalent reaction was more favorable for the production of polymeric molecules.

### 3.3. High-Performance Liquid Chromatography Analysis

As shown in Figure 1E, the elution peaks of GL and US-GL appeared at 24.1453 min and 24.1442 min, respectively, revealing that US had no significant effect on the molecular weight of GL. Comparatively, the elution time of GLE reduced to 24.06201, which showed that EGCG covalently reacted with GL to produce conjugates of greater molecular weight. Li et al. [28] reported that the elution peak retention times of both chlorogenic acid/gliadin and lutenolin/gliadin conjugates were significantly shorter compared to the native gliadin. The elution time of US-GLE was shorter than that of GLE. Necessarily, more EGCG was coupled to GL in the US/hydrogen peroxide/ascorbic acid environment, thus increasing the molecular mass. This was consistent with the above SDS-PAGE analysis.

### 3.4. Phenol Content in Conjugates

In view of the shortcomings of SDS-PAGE and HPLC, the EGCG conjugation efficiency was evaluated. The EGCG content in GLE was 31.3 mg/g, while the EGCG content in US-GLE increased to 46.38 mg/g. The proteins had a low level of aggregation after the strong shearing effect provided by US, such as the case of the fava bean protein (1200 W, 10 and 20 min, 20 KHz) and fish MPN (400 W, 10 min, 20 KHz). In this regard, more recognition sites were exposed, allowing the dynamic reaction equilibrium to proceed in a direction that was conducive to the formation of conjugates. In the ultrasound environment, the exposition of hydrophobic groups may enhance the adsorption of polyphenols by proteins. [29]. Polyphenols were more likely to form covalent bonds with hydrophobic proteins in oxidizing environments (e.g., alkaline environments, or those with free radicals or enzymes). Furthermore, previous work found that the extra OH^-^ generated by US could also affect the covalent reaction of GL [9]. Li et al. [30] similarly found that more conjugates were produced in a US environment. Therefore, the ultrasound environment was more favorable for the covalent reaction.

### 3.5. UV Spectroscopic Analysis

**Figure 2:**
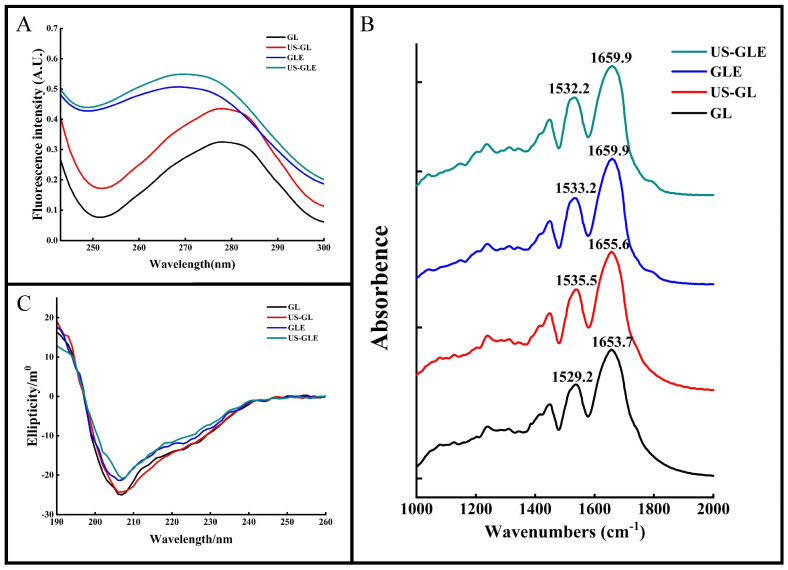
Structural analysis of samples. Ultraviolet absorption (UV-Vis) (**A**), Fourier transform infrared (FT-IR) (**B**) and circular dichroism (CD) (**C**) spectra of GL, US-GL, GLE and US-GLE.

The aromatic amino acids (tyrosine, tryptophan, phenylalanine, etc.) in proteins have a characteristic UV absorption at 270–280 nm. The intensity and position of the absorption peaks can be used to characterize changes in the microenvironment of aromatic amino acids. As shown in Figure 2A, the UV absorption peak of US-GL was significantly enhanced. The increase in aromatic amino acids exposed to the protein surface after ultrasound treatment changed its molar extinction coefficient. The UV absorption peaks of GLE and US-GLE were greatly enhanced and accompanied by a blue shift. This result showed that the covalent reaction drastically changed the GL spatial structure, causing a change in the microenvironment of the aromatic amino acids. US-GLE had stronger absorption and a farther blue shift than GLE. Evidently, the synergistic effect of ultrasonic cavitation and EGCG increased the GL spatial structure’s transition, which promoted the electronic conversion and energy level difference.

### 3.6. Protein Secondary Structure Analysis

FT-IR was used to analyze changes in the structure of the GL molecules (Figure 2B). The Amide I band (1600–1700 cm^−1^) and the Amide II band (1500–1600 cm^−1^) correspond to the C=O and the C-N stretch, respectively. These positions contain information on the secondary structure of the protein. The peak of the Amide I and Amide II bands of GL were 1653.7 cm^−1^ and 1529.2 cm^−1^, respectively. The Amide I and Amide II bands of US-GL red-shifted to 1655.6 cm^−1^ and 1535.5 cm^−1^ revealed the influence of ultrasound on the C=O and C-N groups. The red-shifted Amide I and Amide II bands of GLE and US-GLE indicated the presence of C=O and C-N interactions during the covalent reaction. In this regard, the change in C=O stretching could have been due to the hydrophobic interaction of EGCG with the C-O group, while the C-N stretching might have been due to a covalent linkage of the polyphenol with the protein side chain [31]. Additionally, the Amide I band of the conjugates was more distantly red-shifted than that of US-GL. This result showed that the secondary structure of the protein might show drastic changes in a covalent coupling process.

For the quantitative analysis of the secondary structure of proteins by CD spectroscopy (Figure 2C), a negative band at about 208 nm represents the α-helical structure in GL. The absolute θ values were reduced for all samples compared to GL, which showed that both ultrasound and covalent reactions could disrupt the α-helical structure of GL. As shown in Table 1, the α-helix of US-GL and GLE was reduced by 3.62% and 3.07% compared to GL, respectively. US reduced the α-helix of proteins due to the shear and turbulence it generated that disrupted the intramolecular interactions of the proteins. Similarly, the phenolic hydroxyl groups of the polyphenols in covalent reactions could disrupt hydrogen bonds and thus reduce the helical structure of the proteins [32]. The α-helix of US-GLE was reduced to 15.97%, which indicated that the covalent reaction in the ultrasonic environment could further destabilize the stable structure in GL, contributing to the formation of the loose spatial structure. Regarding protein structure, the intra-chain disulfide bonds of GL were broken by ultrasound to facilitate the expansion of the spatial structure, which was favored by a further modification of the polyphenols, leading to a further reduction in the ordered helical structure [33]. Research has found that the phenolic hydroxyl and benzene ring promote non-covalent interactions between proteins and polyphenols [34], thus limiting the expansion of protein structures. However, the ultrasound environment increased the exposure of reactive groups (-SH, -NH_2_, and proline) in the protein chain structure, resulting in increased molecule sensitivity. Collectively, the chain structure in the ultrasound environment had a more flexible character and was more conducive to forming covalent bonds with polyphenols.

### 3.7. Microstructure Observation

**Figure 3:**
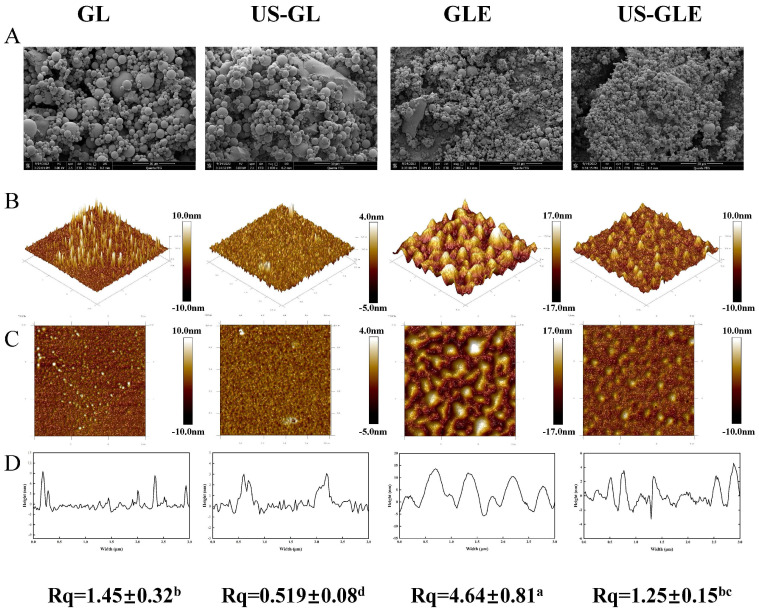
Results of microstructure observation samples. Scanning electron microscopy (SEM, Magnification of 5000 times) (**A**), 3D distribution image (**B**), 2D distribution image (**C**), cross section (**D**) and surface roughness (Rq) of samples, in observation of GL, US-GL, GLE, and US-GLE. “±” refers to the standard deviation. Values with different letters (a–d) in the figure indicate significant differences (*p* < 0.05).

The microstructure of GL was characterized by SEM (Figure 3A). GL appeared as spheres of different sizes, with polymeric structures formed between molecules through hydrogen and hydrophobic bonds. The degree of aggregation of GL was destroyed by a ultrasonic cavitation effect, presenting a homogeneous dispersion. GLE and US-GLE showed tiny particles, revealing that EGCG covalent grafting decreased the non-covalent interactions between GL. He et al. [23] found that EGCG significantly reduced the degree of aggregation of Ara-h1 and formed small spherical particles. As expected, the aggregation effect of the protein was disrupted by the cavitation shear, which provided favorable conditions for the protein–polyphenol covalent reaction.

Atomic force microscopy (AFM) was used to analyze the surface structure of biological materials (Figure 3B). The GL surface showed an irregular spherical shape. With the application of US, GL appeared to be a mesh-like structure. Meanwhile, the height of the cross-section of US-GL was reduced, showing a more uniform planar distribution. This was because the high-intensity mechanical effects of US disrupted the structure of the protein macromolecules. Research has suggested that gluten forms a reticular structure through intramolecular or intermolecular disulfide bonding [35]. However, this was not found in this study because GL was low in sulfhydryl, which meant that it did not readily form disulfide bonds. The appearance of the reticular structure after US may be due to the unfolding of the protein structure and the oxidation of sulfhydryl groups. GL changed from a spherical distribution to an irregular cluster structure after covalent reaction. It was noteworthy that the surface of US-GLE showed a spherical shape similar to that of GL, and its cross-sectional height and Rq were significantly reduced. It was assumed that the GL/EGCG covalent reaction proceeded simultaneously with the local non-covalent reaction in this conventional free radical environment. Hydrogen bonding, van der Waals forces, and dipole/dipole interactions enhanced the binding between the complexes [36], of which the resulting unstable large granule folding limited the contact of the polyphenols with the proteins [37]. Comparatively, the ultrasonic environment limited the creation of such aggregates [38], although weak aggregation between conjugates may have been unavoidable. In another regard, the reticular structure presented by GL in the ultrasound environment prior to contact with EGCG might have been more favorable for the adsorption of EGCG particles. However, the cavitation effect and the covalent modification were completed quickly. Therefore, the reticular structure was not found in US-GLE. US-GLE had an advantage over GLE in terms of structural flexibility. It can be confirmed that the alteration of the GL microstructure as well as the mode of interaction with EGCG in the ultrasonic environment enhanced the effect of the oxidative modification of the free radicals.

### 3.8. Surface Hydrophobicity Analysis (SHY)

**Figure 4:**
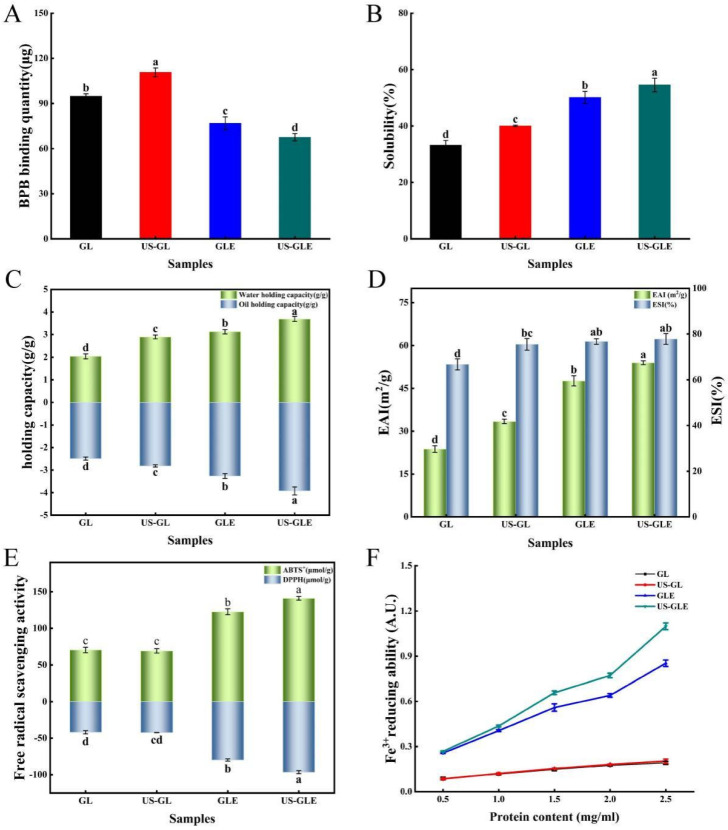
Surface hydrophobicity (**A**), solubility (**B**), water/oil holding capacity (**C**), emulsification/emulsification stability (**D**), ABTS+/DPPH free radical scavenging properties (**E**), and reducing power of Fe^3+^ (**F**) analysis of GL, US-GL, GLE, and US-GLE. Values with different letters (a–d) in the figure indicate significant differences (*p* < 0.05).

Bromophenol blue binding (BPB) was used to indicate the SHY of the protein, in which a higher BPB indicated the stronger SHY of the protein. SHY reflects the amount of exposed hydrophobic groups in proteins, which plays a critically important role in protein interface-related properties. As shown in Figure 4A, ultrasound treatment increased the BPB binding of GL by 15.86 μg. This was mainly due to shear, turbulence and cavitation, which disrupted and rearranged some of the protein aggregates as well as the spatial shape of the protein itself. Eventually, the hydrophobic cavity of the protein was exposed, and the hydrophobic molecules started to transfer. The BPB content of GLE reduced to 76.87 μg, which was 17.99 μg lower than that of GL, showing that the covalent modification of EGCG masked the hydrophobic residues of the protein. This was understandable, since some hydrophobic groups on the GL surface were consumed by covalent coupling with EGCG. Meanwhile large amounts of phenolic hydroxyl groups were also introduced during the reaction, thus further inhibiting thus further inhibiting the SHY of the protein. Liu et al. [39] also found that polyphenols with high phenolic hydroxyl content had a stronger inhibitory effect on protein hydrophobicity. In contrast, Pi et al. [22] reported that the SHY of soy protein was significantly increased after covalent reactions with gallic acid, caffeic acid and tannic acid. The change in correlation between phenolic modification and SHY for proteins was influenced by the characteristics of polyphenols, proteins, and the ratio of substances reacting with polyphenols and proteins. Remarkably, the BPB binding of US-GLE was only 67.55 μg, which was decreased by 9.31 μg compared to GLE. The fundamental reason was the higher content of EGCG in US-GLE. Although ultrasound treatment exposed hydrophobic groups within the GL, these additional added groups were consumed by the enhanced covalent reaction. Some studies have suggested that EGCG has strongly steric hindrance owing to the presence of many benzene rings and phenolic hydroxyl groups in the structure [10]. Therefore, US-GLE was subjected to a greater spatial site resistance to EGCG than GLE was, and such site resistance limited the exposure of hydrophobic cavities and the transfer of hydrophobic groups from US-GLE.

### 3.9. Solubility Analysis

Solubility is the basic functional property of proteins in food processing, and it directly affects the performance of other functional properties of proteins. As shown in Figure 4B, the solubility of US-GL increased to 40.06%. The GL was cleaved into smaller particles after US, which increased the specific surface area of the protein, resulting in enhanced interaction with water. GLE was better than US-GL in water solubility. The reason was mainly that the -OH in the covalent complex has hydrophilic, which enhanced the hydration of the protein molecules [40]. Moreover, the hydrophobic amino acid residues in GL might have been consumed as polyphenol linkage sites during the covalent reaction, thus weakening the hydrophobicity of GL. The solubility of US-GLE was 16.92% higher than that of GL and 4.42% higher than that of GLE. Since the content of EGCG in US-GLE was higher than that of GLE, the improvement in its hydration performance by -OH was more significant. Furthermore, EGCG covered the surface hydrophobic group in GL through covalent bonding, which enhanced the hydrophilicity while inhibiting the hydrophobicity of GL. The US suppressed the weak interactions between the conjugates, while stretching their spatial structure. This made it easier for it to disperse in the aqueous phase, which was beneficial for the improvement of solubility.

### 3.10. Water and Oil Holding Capacity (Whc and Ohc) Analysis

Water-holding capacity is an important property of proteins during processing. Protein molecules were combined with water by electrostatic force between the polar groups on the surface and water molecules, or water molecules were attracted by the hydrophilic groups of the protein to combine with it. As shown in Figure 4C, the Whc of GL was significantly enhanced after ultrasound treatment. The main reason was that the disruption of intramolecular hydrogen bonds led to a looser GL structure, making it easier to encapsulate water molecules. As found by AFM, the reticular structure formed after the US also benefited the Whc of the protein. Furthermore, the translocation of polar amino acid groups to the surface can also enhance the interactions of proteins with water [41]. The improvement in the Whc of GLE mainly originated from the reduction of protein particles, which can be supported by SEM, as the small size of the protein particles facilitated the exposure of polar amino acid side chains [42]. Another explanation was that EGCG masked the hydrophobic amino acid residues during the covalent reaction, thus making the conjugated molecules more accessible to water molecules [43]. Notably, the water-holding capacity of US-GLE was 0.57 g/g higher than that of GLE. This could be explained by the presence of intermolecular disulfide bond cross-linking, contributing to the formation of the GL intermolecular network structure. The network structure benefited protein water retention. Alternatively, the agglomerated structure of the conjugates was disrupted by ultrasound, which might have been more favorable for its binding to water molecules.

The Ohc of GL increased from 2.48 g/g to 2.81 g/g after ultrasound treatment, fundamentally resulting mainly from the change in protein conformation which exposed more lipophilic sites, while the exposure of nonpolar groups and hydrophobic regions enhanced the interaction between the protein molecules and lipids [44]. The Ohc values of GLE and US-GLE was 0.78 g/g and 1.44 g/g, which are higher than those of GL, respectively. In this regard, the conjugates had a larger specific surface area, thereby potentially possessing more lipophilic sites.

### 3.11. Emulsifying Capacity Analysis

Proteins have been often used in food processing as emulsifiers to prevent the deterioration of food properties due to their amphiphilic nature and film-forming ability. As shown in Figure 4D, the EAI and ESI of GL increased by 9.70 m^2^/g and 8.75%, respectively, after US, indicating that the ultrasound enhanced emulsion formation. This result suggested that ultrasound promotes the exposure of hydrophobic regions hidden inside the GL structure. More protein molecules can be adsorbed at the water–oil interface.

The EAI and ESI of conjugates were improved more substantially, where EAI of GLE and US-GLE were 23.89 m^2^/g and 30.24 m^2^/g higher than GL. Research has shown that the reduced protein particle size allowed easier dispersion at the water–oil interface, enhancing emulsification performance [45]. The improvement in emulsification performance of the conjugate was probably due to the advantage of particle size, as seen from the SEM analysis. Furthermore, the covalent modification increased the interfacial viscoelasticity and spatial repulsion of the emulsions, making it more stable, making it more stable [46]. Notably, the EAI of US-GLE was improved by 6.35 m^2^/g compared to GLE. Welc et al. [47] reported that phenolics could cut the intra-strand disulfide bonds, which maintain the conformational stability of proteins, thus conferring a more flexible spatial structure. Moreover, ultrasound treatment can also break the intra-strand disulfide bond of GL. Therefore, the synergistic effect of cavitation and EGCG coupling conferred a more flexible molecular structure to GL. The increase in irregular convolutions raised the thermodynamic adsorption at the oil–water interface and the spatial repulsion between emulsions [48]. In some research [49], the emulsification properties of proteins were positively correlated with the content of hydrophobic groups because the exposure of hydrophobic groups can increase the adsorption of protein molecules at the water–oil interface [50]. However, this was not found in the present study. The essential reason was that protein spatial conformation determined the adsorption rate and recombination ability in the water–oil cross section. Therefore, the enhancement in the emulsification performance of US-GLE was due to the modification of the structure by the synergistic effect of US and EGCG, which made the hydrophilic/hydrophobic equilibrium easier to reach.

### 3.12. Antioxidant Activity Analysis

The antioxidants of GL and GLE were analyzed mainly by ABTS, DPPH radical scavenging activities and Fe^3+^ reduction capacities (Figure 4E,F). GL showed weak free radical scavenging and ion-reducing abilities due to the antioxidant peptides in its structure. The antioxidant properties of US-GL were not significantly changed compared to those of GL, showing that the ultrasound could not produce an effective improvement in the antioxidant properties of GL. Relatively, the free radical scavenging ability of GLE was substantially enhanced, which was mainly attributed to the phenolic hydroxyl group in the GLE backbone. Research has shown that the antioxidant properties of phenols depend on the number of phenolic hydroxyl groups in their chemical structure [7]. He et al. [23] reported that Ara h1-EGCG had a stronger free radical scavenging capacity than Ara h1-CA (chlorogenic acid) did due to the higher content of phenolic hydroxyl groups in EGCG. Xu et al. [51] also found that polyphenols with higher phenolic hydroxyl content were more beneficial in improving the antioxidant properties of the conjugated system. Another possibility was that parts of the reducing products were generated during the free radical-induced covalent reaction to interact with GL, thus conferring its reducing ability [52].

The values of the ABTS and DPPH of US-GLE (140.99 μmol/g, 96.61 μmol/g) were significantly higher than those of GL (70.44 μmol/g, 41.85 μmol/g) and GLE (122.62 μmol/g, 79.87 μmol/g). Noteworthily, US-GLE contained a higher phenol content than GLE. In other words, the phenolic hydroxyl content of the US-GLE backbone was higher. In this regard, the reduction-active groups in EGCG and the spatial characteristics that interacted with the macromolecule (adjacent/relative) were preserved under the ultrasound environment. Meanwhile, free radical oxidation and the sonocavitation effect act synergistically to increase the load of antioxidant molecules in proteins. The analysis of the antioxidant properties corroborated the previous conjecture. US did not significantly affect the antioxidant properties of GL; therefore, the loading of the antioxidant groups within the system determined the antioxidant properties of the conjugates. The results suggested that the combination of US and free radical oxidation was more effective at increasing antioxidant activity.

### 3.13. Digestibility Analysis

**Figure 5:**
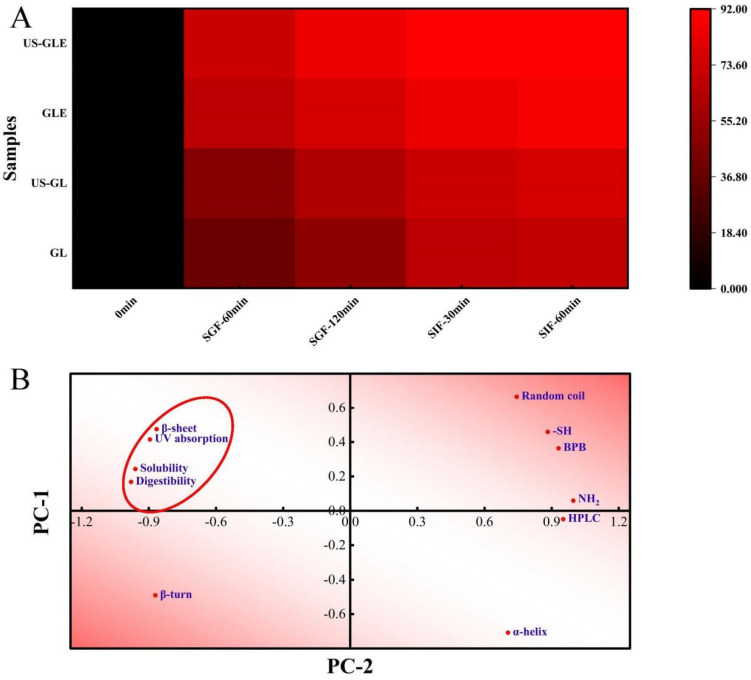
Heat map of digestibility with time (**A**). Principal component analysis (**B**) was performed on the secondary structure (α-helix, β-fold, β-turn, and random coil), UV-vis wavelength scan (UV absorption), group content (-SH and -NH_2_), exposure of hydrophobic group (BPB), HPLC elution time (HPLC) and final digestibility (digestibility) of the previously described samples, with PC-1 accounting for 79.87% of the data and PC-2 accounting for 18.35% of the data. The red ellipse indicates the clustering of physicochemical, structural characteristics with digestibility.

Protein digestibility affects the body’s utilization of amino acids in food, and is primarily influenced by the spatial conformation of proteins. This work analyzed the changes in the digestive properties of GL by simulating digestion with gastric/intestinal fluids (Figure 5A). US had a weak improvement on the digestibility of GL, with the digestibility of US-GL being 5.64% higher than that of GL at SIF-60 min. The improvement in the digestibility of GL by ultrasound treatment was mainly concentrated in the gastric juice digestion phase, but less effective in the intestinal fluid digestion phase. Cavitation and shearing stretched the protein’s conformation. The change in molecular structure-exposed enzyme cleavage sites (relevant sites include aromatic amino acids, proline and arginine) were more susceptible to recognition by pepsin. The GL spatial conformation was almost completely disrupted at the end of SGF. However, the remaining digestive-resistant structures or peptide segments could not be fully consumed in SIF. The digestibility of GE was significantly higher than that of GL and US-GL. More than 70% of GE was consumed in the SGF stage, while less than 20% of that was hydrolyzed at the SIF stage. The digestibility of US-GLE reached 84.74% at SGF-120 min. This showed that the US and EGCG covalent reaction made it easier to expose the internal pepsin action site of G, thus improving the digestibility.

The correlation between the structure, physicochemical properties, and digestibility of GL was further analyzed. As shown in Figure 5B, the digestibility positively correlated with the UV absorption peak intensity, water solubility, and β-folding, while it negatively correlated with the α-helix. Proteins with strong solubility were easier to disperse uniformly in the aqueous phase, which made it convenient for proteases to make contact with them. GL with strong UV absorption had high digestibility, which confirmed the previous speculation that the exposed aromatic amino acids were involved in the hydrolysis as enzymatic sites of action. The α-helix maintained the stability of the secondary structure so that the reduction of its content was also more beneficial to protein digestibility. Furthermore, the elution time of the HPLC peaks was negatively correlated with the digestibility, which further confirmed the improved effect of EGCG on GL digestibility.

### 3.14. Principles of Different Reaction Systems

**Figure 6:**
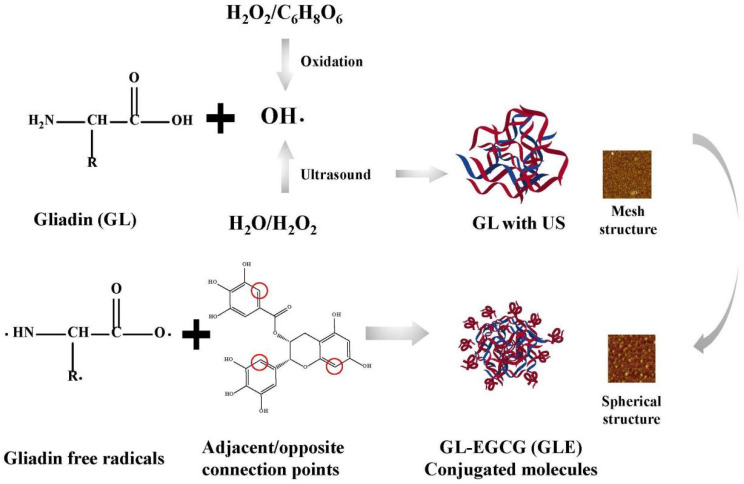
US on the mechanism of the reinforcing effect of the covalent reaction.

In this study, US environment-enhanced EGCG covalent modifications were discussed according to the following inferences (Figure 6). In fact, the free radical-induced covalent reaction determinant was OH^-^, which was where the US synergistic mechanism lay. These ultimately led to the further oxidation of protein side chains, which was the production of protein-free radicals. Despite the significant OH^-^ oxidation, the changes in the structure of the protein itself cannot be ignored. The conclusion showed a reticulated and spherical structure that also verifies the effect of the ultrasound environment on the protein surface. This structure made it more accessible to polyphenol molecules and more susceptible to acoustic cavitation. Additionally, the enhanced water solubility increased the homogeneity of molecules in aqueous protein solutions, and this was the advantage of this reaction system.

## 4. Conclusions

In summary, the cavitation effect in the ultrasonic environment induced the production of more OH^-^ within the system, which intensified the oxidation of GL to form more protein radicals. Meanwhile, the shear, turbulent effect of the ultrasound boosted the flexibility of the protein molecular structure, including the exposition of hydrophobic groups, the reduction in the α-helix and the loosening of the tertiary structure. The polyphenol equivalent of the conjugates was increased to 46.38 mg/g due to the ultrasound-assisted effect. The ultrasonic treatment destroyed the cluster structure of the conjugated molecule, further reducing the helical structure within the conformation. Ultrasound improved GL processing properties (water solubility, water/oil holding and emulsification). These were mainly attributed to the modification of the protein structure and the facilitation of covalent reactions by the US. Moreover, ultrasound-assisted free radical oxidation enhanced the loading of antioxidant molecules in the conjugate. The synergistic effect of ultrasound and polyphenol modifications also enhanced the digestibility of GL. This study explained the potential mechanism of the synergistic effect of US and free radical oxidation on the structural changes in GL, providing the possibility of expanding the application of covalent modifications in the food industry.

## Figures and Tables

**Table 1:** Content of each component in samples.

Samples	GL	US-GL	GLE	US-GLE
Polyphenol equivalents (mg/g protein)	-	-	31.30 ± 0.46 ^b^	46.38 ± 0.79 ^a^
α-helix(%) by CD	20.98 ± 0.77 ^a^	17.36 ± 0.56 ^b^	17.91 ± 0.22 ^b^	15.97 ± 0.45 ^c^
β-sheet(%) by CD	40.57 ± 1.16 ^c^	42.36 ± 0.77 ^b^	42.61 ± 0.88 ^b^	44.54 ± 0.45 ^a^
β-turn(%) by CD	19.78 ± 0.40 ^b^	16.84 ± 0.05 ^c^	21.76 ± 0.26 ^a^	22.73 ± 1.35 ^a^
Random coil(%) by CD	18.67 ± 0.64 ^b^	23.15 ± 0.69 ^a^	17.73 ± 0.67 ^bc^	16.76 ± 0.52 ^c^

Note: all data is expressed by mean ± SD. Different letters in the same line represent significant differences (*p* < 0.05) from each other. In the table, - means not detected in the sample. CD: circular dichroism.

## Data Availability

The data presented in this study are available on request from the corresponding author.

## References

[1] Domenek S., Brendel L., Morel M.-H., Guilbert S. (2004). Swelling Behavior and Structural Characteristics of Wheat Gluten Polypeptide Films. Biomacromolecules.

[2] Abedi E., Pourmohammadi K. (2021). Chemical modifications and their effects on gluten protein: An extensive review. Food Chem..

[3] Joye I.J., Lagrain B., Delcour J.A. (2009). Use of chemical redox agents and exogenous enzymes to modify the protein network during breadmaking—A review. J. Cereal Sci..

[4] Wieser H. (2007). Chemistry of gluten proteins. Food Microbiol..

[5] Ng S.W., Lu P., Rulikowska A., Boehm D., O’Neill G., Bourke P. (2021). The effect of atmospheric cold plasma treatment on the antigenic properties of bovine milk casein and whey proteins. Food Chem..

[6] Yao Y., Jia Y., Lu X., Li H. (2022). Release and conformational changes in allergenic proteins from wheat gluten induced by high hydrostatic pressure. Food Chem..

[7] Quan T.H., Benjakul S., Sae-leaw T., Balange A.K., Maqsood S. (2019). Protein-polyphenol conjugates: Antioxidant property, functionalities and their applications. Trends Food Sci. Technol..

[8] Xue F., Li C., Adhikari B. (2020). Physicochemical properties of soy protein isolates-cyanidin-3-galactoside conjugates produced using free radicals induced by ultrasound. Ultrason. Sonochem..

[9] Chen J., Chen X., Zhou G., Xu X. (2022). New insights into the ultrasound impact on covalent reactions of myofibrillar protein. Ultrason. Sonochem..

[10] Chen J., Zhang X., Fu M., Chen X., Pius B.A., Xu X. (2021). Ultrasound-assisted covalent reaction of myofibrillar protein: The improvement of functional properties and its potential mechanism. Ultrason. Sonochem..

[11] Liu J., Song G., Yuan Y., Zhou L., Wang D., Yuan T., Li L., He G., Yang Q., Xiao G. (2022). Ultrasound-assisted assembly of β-lactoglobulin and chlorogenic acid for non-covalent nanocomplex: Fabrication, characterization and potential biological function. Ultrason. Sonochem..

[12] Ma L., Li A., Li T., Li M., Wang X., Hussain M.A., Qayum A., Jiang Z., Hou J. (2020). Structure and characterization of laccase-crosslinked α-lactalbumin: Impacts of high pressure homogenization pretreatment. LWT-Food Sci. Technol..

[13] Wu X., Lu Y., Xu H., Lin D., He Z., Wu H., Liu L., Wang Z. (2018). Reducing the allergenic capacity of β-lactoglobulin by covalent conjugation with dietary polyphenols. Food Chem..

[14] Xu Y., Dong M., Tang C., Han M., Zhou G. (2019). Glycation-induced structural modification of myofibrillar protein and its relation to emulsifying properties. LWT-Food Sci. Technol..

[15] Rawel H.M., Rohn S., Kruse H.-P., Kroll J. (2002). Structural changes induced in bovine serum albumin by covalent attachment of chlorogenic acid. Food Chem..

[16] Zhang Y., Liu P., Wang C., Zhang F., Linhardt R.J., Eliezer D., Li Q., Zhao J. (2022). Homogalacturonan from squash: Characterization and tau-binding pattern of a sulfated derivative. Carbohydr. Polym..

[17] Lin X., Ye L., He K., Zhang T., Sun F., Mei T., Wu X. (2022). A new method to reduce allergenicity by improving the functional properties of soybean 7S protein through covalent modification with polyphenols. Food Chem..

[18] Li T., Wang L., Geng H., Zhang X., Chen Z. (2021). Formation, structural characteristics, foaming and emulsifying properties of rice glutelin fibrils. Food Chem..

[19] Tang Y., Yang Y., Wang Q., Tang Y., Li F., Zhao J., Zhang Y., Ming J. (2019). Combined effect of carboxymethylcellulose and salt on structural properties of wheat gluten proteins. Food Hydrocoll..

[20] Zhang J., Liu Q., Chen Q., Sun F., Liu H., Kong B. (2022). Synergistic modification of pea protein structure using high-intensity ultrasound and pH-shifting technology to improve solubility and emulsification. Ultrason. Sonochem..

[21] Zhang S., Zheng L., Zheng X., Ai B., Yang Y., Pan Y., Sheng Z. (2019). Effect of steam explosion treatments on the functional properties and structure of camellia (Camellia oleifera Abel.) seed cake protein. Food Hydrocoll..

[22] Pi X., Liu J., Sun Y., Ban Q., Cheng J., Guo M. (2023). Protein modification, IgE binding capacity, and functional properties of soybean protein upon conjugation with polyphenols. Food Chem..

[23] He W., Zhang T., Velickovic T.C., Li S., Lyu Y., Wang L., Yi J., Liu Z., He Z., Wu X. (2020). Covalent conjugation with (−)-epigallo-catechin 3-gallate and chlorogenic acid changes allergenicity and functional properties of Ara h1 from peanut. Food Chem..

[24] Li T., Bu G., Xi G. (2021). Effects of heat treatment on the antigenicity, antigen epitopes, and structural properties of β-conglycinin. Food Chem..

[25] Liu F., Ma C., Gao Y., McClements D.J. (2016). Food-Grade Covalent Complexes and Their Application as Nutraceutical Delivery Systems: A Review. Compr. Rev. Food Sci. Food Saf..

[26] Zhang K., Wen Q., Li T., Wang Y., Zhang Y., Luo D. (2022). Comparative study of the effects of ultrasonic power on the structure and functional properties of gliadin in wheat and green wheat. J. Food Sci..

[27] Liu F., Sun C., Yang W., Yuan F., Gao Y. (2015). Structural characterization and functional evaluation of lactoferrin–polyphenol conjugates formed by free-radical graft copolymerization. RSC Adv..

[28] Zhang K., Wen Q., Li T., Zhang Y., Huang J., Huang Q., Gao L. (2022). Effect of covalent conjugation with chlorogenic acid and luteolin on allergenicity and functional properties of wheat gliadin. J. Cereal Sci..

[29] Sun F., Li B., Guo Y., Wang Y., Cheng T., Yang Q., Liu J., Fan Z., Guo Z., Wang Z. (2022). Effects of ultrasonic pretreatment of soybean protein isolate on the binding efficiency, structural changes, and bioavailability of a protein-luteolin nanodelivery system. Ultrason. Sonochem..

[30] Li Z., Zheng Y., Sun Q., Wang J., Zheng B., Guo Z. (2021). Structural characteristics and emulsifying properties of myofibrillar protein-dextran conjugates induced by ultrasound Maillard reaction. Ultrason. Sonochem..

[31] Geng M., Feng X., Yang H., Wu X., Li L., Li Y., Teng F. (2022). Comparison of soy protein isolate-(−)-epigallocatechin gallate complexes prepared by mixing, chemical polymerization, and ultrasound treatment. Ultrason. Sonochem..

[32] Liu J., Song G., Zhou L., Yuan Y., Wang D., Yuan T., Li L., He G., Xiao G., Chen F. (2023). Sonochemical effects on fabrication, characterization and antioxidant activities of β-lactoglobulin-chlorogenic acid conjugates. Ultrason. Sonochem..

[33] Pi X., Sun Y., Liu J., Peng Z., Liang S., Cheng J., Jiang Y. (2022). Multi-spectral and proteomic insights into the impact of proanthocyanidins on IgE binding capacity and functionality in soy 11S protein during alkali-heating treatment. Int. J. Biol. Macromol..

[34] Zhao X., Xu X., Zhou G. (2021). Covalent chemical modification of myofibrillar proteins to improve their gelation properties: A systematic review. Compr. Rev. Food Sci. Food Saf..

[35] Zhang Y., Wang B., Zhou C., Atungulu G.G., Xu K., Ma H., Ye X., Abdualrahman M.A. (2016). Surface topography, nano-mechanics and secondary structure of wheat gluten pretreated by alternate dual-frequency ultrasound and the correlation to enzymolysis. Ultrason. Sonochem..

[36] Chen J., Gao Q., Zhou G., Xu X. (2022). Interactions between the protein-epigallocatechin gallate complex and nanocrystalline cellulose: A systematic study. Food Chem..

[37] Zhou S.-D., Lin Y.-F., Xu X., Meng L., Dong M.-S. (2020). Effect of non-covalent and covalent complexation of (−)-epigallocatechin gallate with soybean protein isolate on protein structure and in vitro digestion characteristics. Food Chem..

[38] Alavi F., Chen L., Emam-Djomeh Z. (2021). Effect of ultrasound-assisted alkaline treatment on functional property modifications of faba bean protein. Food Chem..

[39] Liu X., Song Q., Li X., Chen Y., Liu C., Zhu X., Liu J., Granato D., Wang Y., Huang J. (2021). Effects of different dietary polyphenols on conformational changes and functional properties of protein–polyphenol covalent complexes. Food Chem..

[40] Xu H., Zhang T., Lu Y., Lin X., Hu X., Liu L., He Z., Wu X. (2019). Effect of chlorogenic acid covalent conjugation on the allergenicity, digestibility and functional properties of whey protein. Food Chem..

[41] Zhang H., Chen G., Liu M., Mei X., Yu Q., Kan J. (2020). Effects of multi-frequency ultrasound on physicochemical properties, structural characteristics of gluten protein and the quality of noodle. Ultrason. Sonochem..

[42] Wu W., Hua Y., Lin Q., Xiao H. (2011). Effects of oxidative modification on thermal aggregation and gel properties of soy protein by peroxyl radicals. Int. J. Food Sci. Technol..

[43] Ozdal T., Capanoglu E., Altay F. (2013). A review on protein–phenolic interactions and associated changes. Food Res. Int..

[44] Higuera-Barraza O., Del Toro-Sanchez C., Ruiz-Cruz S., Márquez-Ríos E. (2016). Effects of high-energy ultrasound on the functional properties of proteins. Ultrason. Sonochem..

[45] Lam R.S.H., Nickerson M.T. (2013). Food proteins: A review on their emulsifying properties using a structure–function approach. Food Chem..

[46] Sun Y., Zhang S., Xie F., Zhong M., Jiang L., Qi B., Li Y. (2021). Effects of covalent modification with epigallocatechin-3-gallate on oleosin structure and ability to stabilize artificial oil body emulsions. Food Chem..

[47] Welc R., Kłosok K., Szymańska-Chargot M., Nawrocka A. (2022). Effect of chemical structure of selected phenolic acids on the structure of gluten proteins. Food Chem..

[48] Li R., Xiong Y.L. (2023). Disulfide cleavage to improve interfacial behavior and emulsification properties of oat protein. Food Chem..

[49] Kim H.-J., Decker E.A., McClements D.J. (2005). Influence of Protein Concentration and Order of Addition on Thermal Stability of β-Lactoglobulin Stabilized *n*-Hexadecane Oil-in-Water Emulsions at Neutral pH. Langmuir.

[50] Guilmineau F., Kulozik U. (2007). Influence of a thermal treatment on the functionality of hen’s egg yolk in mayonnaise. J. Food Eng..

[51] Xu Y., Han M., Huang M., Xu X. (2021). Enhanced heat stability and antioxidant activity of myofibrillar protein-dextran conjugate by the covalent adduction of polyphenols. Food Chem..

[52] Gu L., Peng N., Chang C., McClements D.J., Su Y., Yang Y. (2017). Fabrication of Surface-Active Antioxidant Food Biopolymers: Conjugation of Catechin Polymers to Egg White Proteins. Food Biophys..

